# Effects of SHINBARO2 on Rat Models of Lumbar Spinal Stenosis

**DOI:** 10.1155/2019/7651470

**Published:** 2019-04-28

**Authors:** So Hyun Park, Ji-Young Hong, Won Kyung Kim, Joon-Shik Shin, Jinho Lee, In-Hyuk Ha, Hwa-Jin Chung, Sang Kook Lee

**Affiliations:** ^1^College of Pharmacy, Natural Products Research Institute, Seoul National University, Seoul 08826, Republic of Korea; ^2^Jaseng Spine and Joint Research Institute, Jaseng Medical Foundation, Seoul 06110, Republic of Korea

## Abstract

Lumbar spinal stenosis (LSS) is a major cause of chronic low back pain; however, only a few therapies which have been used in clinics still have limited effects on functional recovery. SHINBARO2 is a refined traditional formulation for inflamed lesions and relieve pain of muscular skeletal disease. This study aimed at investigating the effects of SHINBARO2 on LSS and at determining its underlying molecular mechanism in rat models. The LSS rat models were set up by surgical operations in 6-week-old male Sprague-Dawley rats. SHINBARO2 was orally or intraperitoneally administered for 14 days. The motor and sensory ability of rats were evaluated using the activity cage and hot plate method. On the termination day, total vertebrae including the disc and spinal cord were excised for ex vivo study. SHINBARO2 improved locomotor functions and pain sensitivity in LSS rat models. Mechanism study suggested that SHINBARO2 inhibited the production of nitric oxide and prostaglandin E_2_ in tissues from LSS-induced rats. SHINBARO2 also suppressed the expression of proinflammatory cytokines including tumor necrosis factor-*α* and interleukin-1*β*. The activation of NF-*κ*B by LSS surgery was effectively reduced by SHINBARO2, which coincided with the inhibition of I*κ*B degradation. In addition, brain-derived neurotrophic factor (BDNF), a potent promoter of neurite growth, and its downstream ERK signaling were also regulated by SHINBARO2. These findings suggest that the effect of SHINBARO2 might be associated in part with the anti-inflammation and pain control in LSS rat models.

## 1. Introduction

Lumbar spinal stenosis (LSS) is one of the major causes of chronic low back pain and is defined as any narrowing of the lumbar spinal canal, nerve root canal, or intervertebral foramina [1]. The most common symptoms of LSS in patients are midline back pain, radiculopathy with neurologic claudication, motor weakness, paresthesia, and impairment of the sensory nerves [2]. LSS is a common degenerative disease in people older than 60 years, and the prevalence of the elderly is expected to be approximately 400,000 in the U.S. [3].

Regarding diagnosis, the degree of LSS confirmed by imaging is less relevant to the actual symptoms. In one cross-sectional study of nonsymptomatic persons aged 60 years or older, only 21% had spinal stenosis [4]. In 1994, the International Association for the Study of Pain (IASP) defined neuropathic pain as “pain initiated or caused by a primary lesion or dysfunction in the nervous system”; however, it is not directly related to the shape of the compressed spinal cord. Therefore, the study of neuropathic pain quantification with the precise mechanism for LSS management is a prerequisite.

Strategies for treating LSS have often focused on relieving symptoms, such as relief from intraspinal pressure, diversification in blood flows, and the metabolism of neural structures, decrease in inflammation, and decompression of the neural elements [5]. The most commonly used methods for treating LSS are surgery and conservative treatment. First, surgical decompression for LSS is usually imposed in severe cases because of the high risk of side effects and financial burden and because of its failure to sustain adequate long-term efficacy. Second, there have been many attempts to use pharmacological management, such as nonsteroidal anti-inflammatory drugs (NSAIDs), gabapentin, opioid analgesics, muscle relaxants, prostaglandins, calcitonin, methylcobalamin, and epidural steroid injections, in conservative treatments. However, the number of studies is limited, and these studies provide low-quality evidence [6].

SHINBARO2 is a refined formulation comprised of nine materials to treat inflamed lesions and relieve pain of muscular skeletal disease: *Paeonia lactiflora* Pall. (Paeoniaceae), *Cibotium barometz* (L.) J. Smith. (Dicksoniaceae), *Saposhnikovia divaricate* Schiskin (Umbelliferae), *Eucommia ulmoides* Oliver (Eucommiaceae), *Acanthopanax sessiliflorum* Seem (Araliaceae), *Achyranthes japonica* Nakai (Amaranthaceae), *Scolopendra subspinipes* mutilans L. Koch (Scolopendridae), *Ostericum koreanum* (Maxim.) Kitagawa (Apiaceae), and *Aralia continentalis* Kitagawa (Araliaceae). Although the specific ingredients of SHINBARO2 have not been completely identified, many studies have reported the pharmacological effects of each materials of SHINBARO2. Previous studies have reported the anti-inflammatory activity of *Eucommia ulmoides* [7] and *Acanthopanax sessiliflorum* [8]. *Achyranthes japonica* also has reported for anti-inflammatory and antiosteoarthritis properties [9]. *Scolopendra subspinipes* mutilans is used primarily to treat joint problems like arthritis, and several experimental approaches have proven its ethnopharmacological activities on inflammatory diseases [10, 11]. *Cibotium barometz* has been widely used in traditional oriental medicine for the treatment of lumbago and rheumatism [12] and has recently been reported on osteoclast formation inhibitory efficacy and bone-strengthening activity [13]. GCSB-5 (SHINBARO) consisted of six crude herbs (*Cibotium barometz*, *Saposhnikovia divaricate*, *Eucommia ulmoides*, *Acanthopanax sessiliflorum*, *Achyranthes japonica*, and *Glycine max*), and five of six components are also included in SHINBARO2. GCSB-5 has been widely used for the treatment of neuropathic and inflammatory diseases such as osteoarthritis [14] and lumbar disc herniation [15]. SHINBARO2 has been administered to musculoskeletal patients in clinics and reported for the most frequently used pharmacopuncture formula to LSS patients in Korea [16]. Although the efficacy and safety of SHINBARO2 pharmacopuncture have also been proven by a randomized controlled trial in hand osteoarthritis and sciatic pain with the lumbar disc herniation [17, 18], the molecular mechanism for the therapeutic effects of SHINBARO2 on LSS has not yet been elucidated.

In this study, we established a minimally invasive rat model of LSS, narrowing the spinal canal by transplanting a silicon tube. This LSS-induced rat model was used to investigate the therapeutic potential of SHINBARO2 by eliminating the improvement of locomotor function and sensibility. Anti-inflammatory and neuroprotective biomarkers were also evaluated by the regulation of protein and mRNA expression. Collectively, these data highlight the potential evidence for the use of SHINBARO2 in the treatment of LSS patients.

## 2. Materials and Methods

### 2.1. Chemicals

Goat anti-rabbit immunoglobulin G- (IgG-) horseradish peroxidase (HRP), goat anti-mouse IgG-HRP, goat anti-goat IgG-HRP, and primary antibodies against iNOS, COX-2, IL-1*β*, NF-*κ*B p65, NF-*κ*B p50, brain-derived neurotrophic factor (BDNF), pro-BDNF, extracellular signal-related kinase (ERK), phospho-ERK (p-ERK), I*κ*B-*α*, phospho-I*κ*B-*α*, and *β*-actin were obtained from Santa Cruz Biotechnology (Santa Cruz, CA, USA). Antibodies against TNF-*α*, MEK, phospho-MEK1/2 and phospho-CREB were purchased from Cell Signaling Technology (Danvers, MA, USA). Gene-specific primers for real-time PCR were synthesized from Bioneer (Daejeon, Korea). The Reverse Transcription Kit was purchased from Promega (Madison, WI, USA). 17*β*-Estradiol (E_2_), hematoxylin, eosin, and other agents unless otherwise indicated were purchased from Sigma-Aldrich (St. Louis, MO, USA).

### 2.2. Preparation of SHINBARO2

Nine crude materials (*Paeonia lactiflora* Pall., *Cibotium barometz* (L.) J. Smith., *Saposhnikovia divaricate* Schiskin, *Eucommia ulmoides* Oliver, *Acanthopanax sessiliflorum* Seem, *Achyranthes japonica* Nakai, *Scolopendra subspinipes* mutilans L. Koch, *Ostericum koreanum* (Maxim.) Kitagawa, and *Aralia continentalis* Kitagawa) were boiled in 70% ethanol for 3 h. The decoction was vacuum-evaporated and refined by an 80% and 90% ethanol-soaking process. The purified extract was then freeze-dried to a powder form. All ingredients were obtained and authenticated by Dr. J. Lee (Jaseng Hospital of Korean Medicine, Seoul, Korea). Voucher specimens of the drugs used in this study are deposited at the herbarium of Jaseng Hospital of Korean Medicine.

### 2.3. Animals

Male Sprague-Dawley (SD) rats (215.1 ± 9.7 g, 6 weeks old) were purchased from the Central Laboratory Animal Inc. (Seoul, Korea) and were grown in the animal care facility at Seoul National University under pathogen-free conditions with a 12 h light-dark schedule. The use and care of animals were carried out in strict accordance with the guidelines of the Seoul National University Institutional Animal Care and Use Committees (IACUC; permission number: SNU150911-1).

### 2.4. In Vivo Spinal Stenosis Model

**Figure 1:**
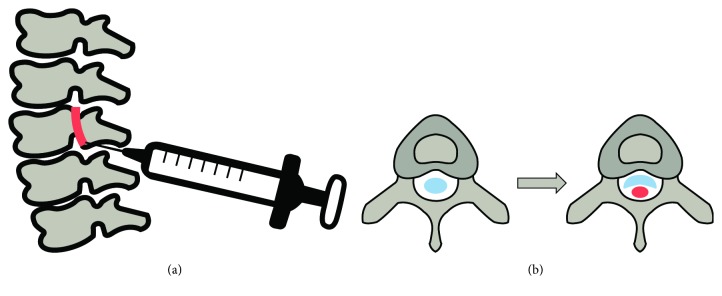
Schemes of LSS-induced surgery in (a) sagittal and (b) transverse planes. A piece of silicone (red) was inserted by a syringe to narrow the spinal canal (white), and the spinal cord was pressurized (blue).

Six-week-old SD rats were anesthetized by intraperitoneal injection of 2,2,2-tribromoethanol in amylene hydrate (250 mg/kg). After confirming the efficacy of anesthetics by toe pinching, the animals were placed in a prone position and shaven to expose their skin of the dorsal spine line. The middle of the spine including the L4 to L5 level was made an incision to expose the ligamentum flavum. Using a 26-gauge syringe, a piece of silicone block (4 × 1 × 1 mm, Bentec Medical Inc., Woodland, CA, USA) was inserted into the epidural space between the L4 and L5 level (Figure 1). The incision was washed with PBS and sutured with polysorb 4 (Medline Industries, Inc., Mundelein, IL, USA) by layer. The rats were returned to their cages with 37°C heating blankets to recuperate after LSS surgery. SHINBARO2 was administered per os (p.o.; 20, 200 mg/kg) or intraperitoneally (i.p.; 2, 10, and 20 mg/kg) to the experimental group once per day for two weeks. Estradiol (E_2_; 1 mg/kg, per os) was used as the positive control drug. All groups were monitored for changes in weight during the experimental period. Following three weeks of drug administration, blood samples were collected from the heart, and tissue samples surrounding the spinal cord were obtained from the 5th lumbar level for further analysis.

### 2.5. Behavioral Test

To evaluate the locomotor activity, we assessed the number of steps using a running wheel. The speed of the wheel was regulated from 4 revolutions per minute (rpm) to 40 rpm over 5 min (JD-A-06A, Jeung Do Bio & Plant Co., LTD., Seoul, Korea). From the time when the rat entered the wheel, the number of walking steps was recorded until the wheel turned 10 laps. The rats were trained to run on the running wheel daily for three days before inducing LSS, and running steps were measured after LSS. Another tool used to estimate responses to pain, such as heat stimulus, was a hot plate analgesia meter (JD-A-10A, Jeung Do Bio & Plant Co., LTD., Seoul, Korea). The 25 cm-diameter plate was set on 52 ± 3°C, and the 30 cm-height cylindrical glass was on the plate. From the beginning of the test, the response time on heat stimulus was estimated when the rats showed nociceptive response such as hind paw licking, hind paw flicking, vocalization, or jumping. Without any reaction, the test was stopped at 60 sec.

### 2.6. Hematoxylin and Eosin (H&E) Staining and Immunohistochemistry Assay

The excised vertebrae were fixed in 4% paraformaldehyde, decalcified in 20% formic acid, and embedded in paraffin. Sectioned slides of the embedded specimens were serially deparaffinized, rehydrated, and stained with hematoxylin and eosin. The morphology was observed and photographed with a Vectra 3.0 Automated Quantitative Pathology Imaging System (PerkinElmer, Waltham, MA, USA). To detect the local expression of BDNF, the tissue samples were reacted with protein kinase K, blocked with blocking solution (10% normal goat serum, 0.1% bovine serum albumin), and exposed to primary antibodies against BDNF and secondary antibody. The samples were then sealed with the cover glass and observed under a Vectra 3.0 Automated Quantitative Pathology Imaging System (PerkinElmer, Waltham, MA, USA).

### 2.7. Measurement of Serum Markers

Blood samples were centrifuged at 1,500 rpm for 10 min. The collected supernatant from the serum samples was frozen at -70°C until further analysis. Serum levels of PGE_2_, TNF-*α*, and IL-1*β* were measured with the corresponding ELISA kits (R&D Systems, Minneapolis, MN, USA). All experiments were conducted per manufacturer's instructions.

### 2.8. Western Blot Assay

Protein samples were collected from the isolated spinal tissue using a protein extraction kit (Active Motif, Carlsbad, CA, USA). The samples were quantified to 10-30 *μ*g and were subjected to 8~15% sodium dodecyl sulfate- (SDS-) polyacrylamide gel at 100 V for 2.5 h. The proteins were transferred onto PVDF membranes (Millipore, Bedford, MA, USA) by electroblotting, and the membranes were blocked for 1 h with blocking buffer (5% bovine serum albumin (BSA) in Tris-buffered saline-0.1% Tween 20 (TBST)) at room temperature. The membranes were then incubated with indicated antibodies (mouse anti-*β*-actin, diluted 1 : 10,000; other antibodies, diluted 1 : 100~1,000 in 5% BSA/TBST) overnight at 4°C and were washed three times for 10 min with TBST. After they were washed, the membranes were incubated with the corresponding secondary antibodies diluted 1 : 2,000 in TBST for 2 h at room temperature and washed three times for 10 mins with TBST. The proteins on the membranes were detected with an enhanced chemiluminescence (ECL) detection kit (LabFrontier, Suwon, Korea) using an LAS-4000 Imager (Fujifilm Corp., Tokyo, Japan).

### 2.9. RNA Isolation and Real-Time Polymerase Chain Reaction (Real-Time RT-PCR)

The total RNA from the tissue was extracted with TRI reagent (Invitrogen, NY, USA) and reverse-transcribed using a Reverse Transcription System (Promega) according to the manufacturer's instructions. Real-time RT-PCR was conducted using iQ™ SYBR^®^ Green Supermix (Bio-Rad, Richmond, CA) according to the manufacturer's instructions. The conditions for the assay were the following: 20 sec at 95°C, 40 cycles of 20 sec at 95°C, 20 sec at 56°C, 30 sec at 72°C, 1 min at 95°C, and 1 min at 55°C. All of the experiments were performed in quadruplicate, and the analysis was performed through the delta delta Cq method [19]. The sequences of the primers are listed in Table 1.

### 2.10. Statistical Analysis

All experiments were repeated at least three times. The data are presented as the means ± standard deviation. The differences between the experimental and control groups were analyzed by one-way analysis of variance (ANOVA). Values of ^∗^*p* < 0.05, ^∗∗^*p* < 0.01, and ^∗∗∗^*p* < 0.001 were considered statistically significant.

## 3. Results and Discussion

### 3.1. Behavioral Assessment and Body Weight

**Figure 2:**
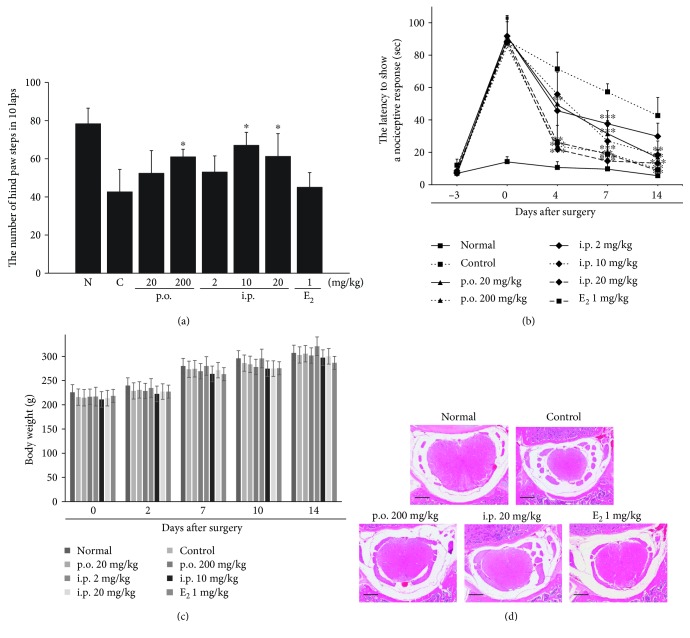
Effects of SHINBARO on locomotor and sensory functions in the LSS rat model. (a) The recovery of locomotor function measured by the running wheel system in the 7th day after LSS operation. (b) Time course of change in response times to a thermal stimulus using a hot plate. (c) Body weight change was monitored during the test period. The data are expressed as the mean ± SD (*n* = 5). (d) Histopathological analysis of the spinal structure following administration of SHINBARO in the LSS rat model. The spinal cord (red) in the spinal canal (white) was stained with hematoxylin and eosin (H&E). Scale bar = 800 *μ*m.

The capacity for locomotion was calculated as the number of hind paw steps during the running wheel test, which was assessed as described in Materials and Methods. The rats in the normal groups without LSS surgery were able to walk on the wheel for 78.3 ± 8.2 steps. The number of steps was significantly decreased in all LSS-induced groups. The SHINBARO2-treated group showed fast improvement in motor function on the 7th day after the LSS operation, whereas the vehicle-treated animals in the control group did show significantly reduced mobility on the running wheel (Figure 2(a)). Moreover, the hot plate test was used to evaluate temperature sensitivity and sensory response activity. Animals were pretrained on the hot plate for 3 days before the experiments began. All animals were able to show a positive response to the thermal stress in 8.3 ± 1.8 sec before the LSS surgery. The latency of nociceptive responses was significantly increased to 88.5 ± 3.7 sec after LSS operation. The animals in the SHINBARO2-treated group started with significantly faster improvement in recognition and reaction to temperature from the 4th day to the endpoint than the vehicle-treated animals in the control group (Figure 2(b)). Additionally, the body weight changes in all groups were monitored over the duration of the experiment to assess possible toxicity of LSS surgery and drug administration. Neither overt toxicity nor a noticeable change in body weight was observed in the rats in SHINBARO2-treated groups compared to the rats in the normal and vehicle control groups (Figure 2(c)). No significant differences were observed among groups; however, a slight delay in body weight gain was observed in the positive control group (E_2_, 1 mg/kg).

### 3.2. Morphology of the Spinal Structure

Transverse sectioned spinal tissue was stained with hematoxylin and eosin (H&E) to observe morphological changes under an optical microscope. The rats in the normal group showed oval-shaped spinal cords and intact spinal canals. However, LSS-induced rats exhibited crushed spinal cords because of the narrowed spinal canals. Administration of SHINBARO2 or E_2_ led to structural recovery of the LSS-induced damage to normal morphology (Figure 2(d)).

### 3.3. Effect of SHINBARO2 on the Expression of iNOS and COX-2

Inducible nitric oxide synthase (iNOS) and cyclooxygenase-2 (COX-2) are well-known inflammatory biomarkers that play an important role in inflammatory processes, and the regulation of this pathway is considered as a proper method for anti-inflammatory treatment. Since local inflammation is a common response to LSS, the inhibitory effects of SHINBARO2 on inflammatory mediators were examined in the serum or tissue from the LSS-stimulated rat model.

**Figure 3:**
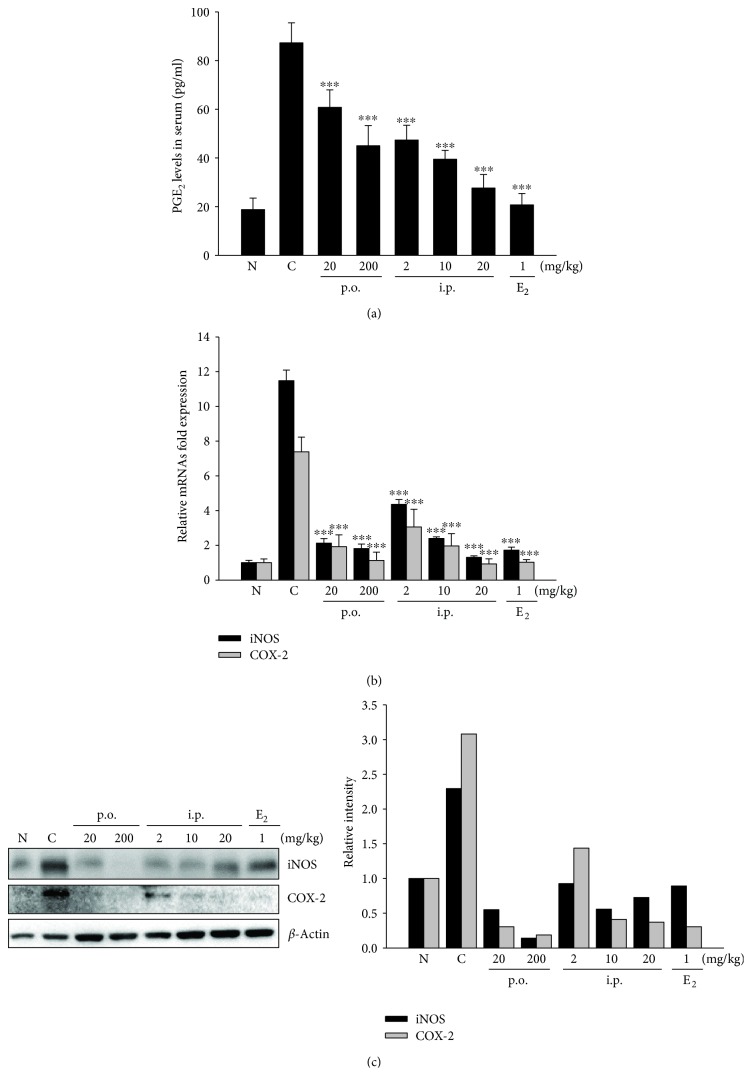
Effects of SHINBARO on inflammatory mediators in the LSS rat model. (a) Serum was collected from rats with LSS and was analyzed for PGE_2_ levels using an ELISA kit. The data are expressed as the mean ± SD (*n* = 4). (b) The mRNA expression of iNOS and COX-2 in the spinal cord was determined by real-time RT-PCR. The results were normalized using *β*-actin as an internal control. The data are expressed as the mean ± SD (*n* = 4). (c) The protein expression of iNOS and COX-2 in spinal cord tissues was determined by western blot analysis. *β*-Actin was used as an internal control. Relative intensity of indicated proteins was semiquantified using the NIH ImageJ software.

To determine whether SHINBARO2 inhibits prostaglandin E_2_ (PGE_2_) production, a PGE_2_ enzyme immunometric assay was used in the serum of LSS-stimulated rats. Serum PGE_2_ was significantly induced in the LSS-operated control group than in the normal unoperated group. SHINBARO2 treatment significantly inhibited the serum PGE_2_ levels in a concentration-dependent manner (Figure 3(a)). To further investigate the effect of SHINBARO2 on the mRNA level of inflammatory mediators, the expressions of iNOS and COX-2 were evaluated by real-time RT-PCR. As shown in Figure 3(b), the expression levels of iNOS and COX-2 were higher in the LSS-induced group than in the normal group. When LSS-induced rats were treated with SHINBARO2 orally or intraperitoneally, the mRNA levels of iNOS and COX-2 were suppressed in comparison to those in the vehicle-treated control group. In addition, the protein expression levels of iNOS and COX-2 were assessed by western blotting, and the expression levels of both iNOS and COX-2 proteins were increased after LSS induction, which was effectively suppressed by SHINBARO2 treatment (Figure 3(c)).

### 3.4. Effect of SHINBARO2 on the Expression of Proinflammatory Cytokines

**Figure 4:**
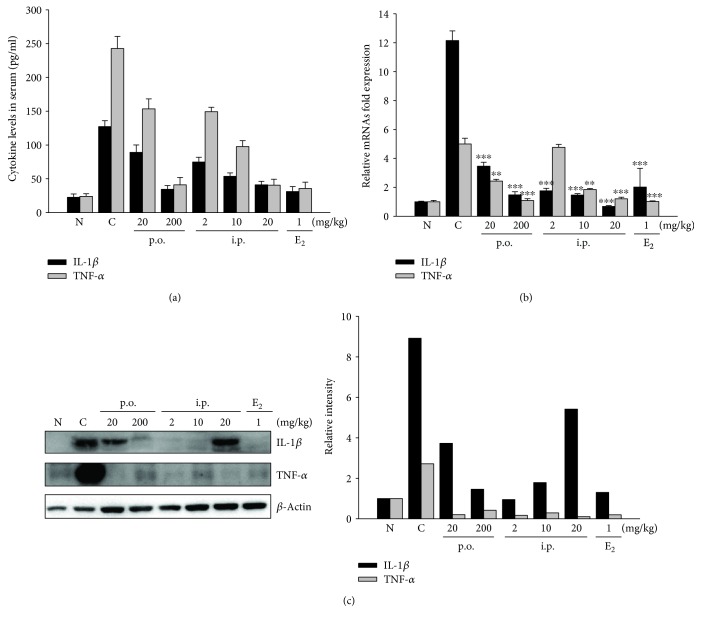
Effects of SHINBARO on proinflammatory cytokines in the LSS rat model. (a) Serum was collected from rats with LSS and was analyzed for IL-1*β* and TNF-*α* levels using an ELISA kit. The data are expressed as the mean ± SD (*n* = 5). (b) The mRNA expression of IL-1*β* and TNF-*α* in a spinal cord tissue was determined by real-time RT-PCR. The results were normalized using *β*-actin as an internal control. The data are expressed as the mean ± SD (*n* = 4). (c) The expression of proinflammatory cytokines in spinal cord tissues was determined by western blot analysis. *β*-Actin was used as an internal control. Relative intensity of indicated proteins was semiquantified using the NIH ImageJ software.

To estimate the regulatory effects of SHINBARO2 on proinflammatory cytokines such as TNF-*α*, IFN-*γ*, and interleukins, the serum and tissue from various groups of rats were collected. The LSS operation increased the levels of TNF-*α* and IL-1*β* in serum, but they were effectively suppressed by SHINBARO2 in a dose-dependent manner (Figure 4(a)). Real-time RT-PCR was used to determine the mRNA expression of TNF-*α* and IL-1*β* in spinal cord tissue. The levels of TNF-*α* and IL-1*β* were higher in the LSS-induced group than in the normal group, and the increased expression in the LSS-induced group was suppressed by SHINBARO2 (Figure 4(b)). Similarly, SHINBARO2 downregulated the proteins of TNF-*α* and IL-1*β* induced by LSS surgery (Figure 4(c)).

### 3.5. Effect of SHINBARO2 on the NF-*κ*B and I*κ*B-*α* Pathways

**Figure 5:**
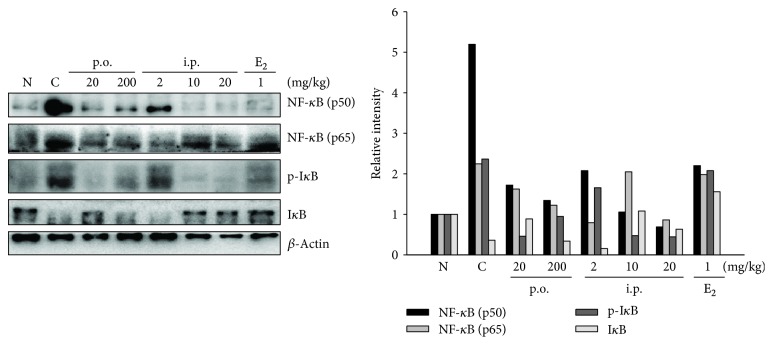
Effects of SHINBARO on the protein levels of NF-*κ*B and I*κ*B-*α* in the LSS rat model. *β*-Actin was used as an internal control. Relative intensity of indicated proteins was semiquantified using the NIH ImageJ software.

NF-*κ*B also plays an essential role in the expression of proinflammatory cytokines as a transcriptional factor [20, 21]. When proinflammatory signals such as iNOS and COX-2 are stimulated, I*κ*B-*α* is phosphorylated by I*κ*B-*α* kinase and degraded via a 26S proteasome-mediated pathway that facilitates NF-*κ*B translocation into the nucleus and thus regulates target gene transcription [22]. Further studies were performed to determine the inhibition of NF-*κ*B transcriptional activity related to the level of NF-*κ*B subunits p65 and p50, spinal cord tissues were collected, and western blot analysis was performed. The protein levels of p65 and p50, subunits of NF-*κ*B, were increased after LSS surgery and downregulated in the SHINBARO2-treated group (Figure 5). Furthermore, the degradation of I*κ*B-*α* by LSS was inhibited by treatment with SHINBARO2. These findings indicate that the anti-inflammatory effect of SHINBARO2 on the LSS rat model is partially associated with the suppression of NF-*κ*B activation.

### 3.6. Effect of SHINBARO2 on the BDNF Signaling Pathway

**Figure 6:**
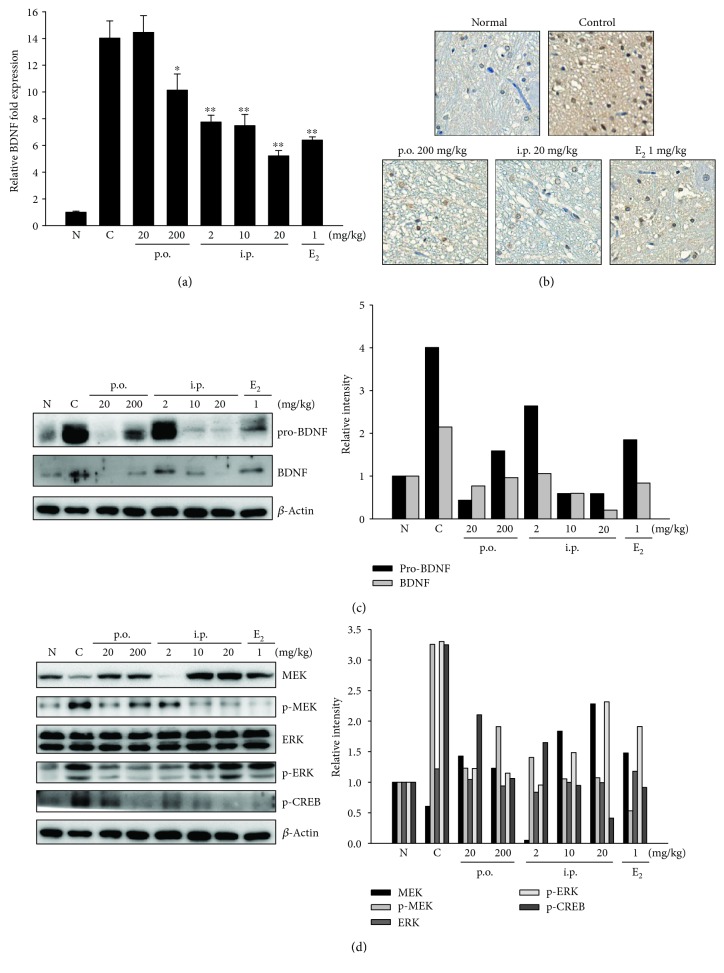
Effects of SHINBARO on the expression of neurotrophic factors in the LSS rat model. (a) The mRNA expression of BDNF in spinal cord tissues was determined by real-time RT-PCR. Total RNA was isolated and further analyzed by real-time RT-PCR. The results were normalized using *β*-actin as an internal control. The data are expressed as the mean ± SD (*n* = 4). (b) Immunohistochemical analysis of BDNF in the spinal cord. (c) Expression of pro-BDNF and BDNF in spinal cord tissues was investigated by western blot analysis. *β*-Actin was used as an internal control. (c) Immunostaining analysis of BDNF-positive cells in the spinal cord. (d) Expression of MEK, p-MEK, ERK, p-ERK, and p-CREB in spinal cord tissues was investigated by western blot analysis. *β*-Actin was used as an internal control. Relative intensity of indicated proteins was semiquantified using the NIH ImageJ software.

Neurotrophin family members are important regulators of neuronal survival, growth, and differentiation. Brain-derived neurotrophic factor (BDNF), a key member of the neurotrophin family, was selected as a biomarker in order to evaluate the neuropathic pain caused by LSS and the beneficial effects of SHINBARO2 administration. To investigate the molecular mechanisms by which SHINBARO2 promotes neuroplasticity and functional recovery relieving pain, we examined the protein and mRNA levels of BDNF in the LSS-induced spinal cord. As shown in Figure 6(a), the mRNA expression of BDNF was increased after LSS induction and was suppressed by SHINBARO2 or E_2_ in a dose-dependent manner. Immunochemical analysis also suggested that the enhanced expression of BDNF by LSS in the spinal cord was significantly suppressed by the treatment of SHINBARO2 as shown in Figure 6(b). In addition, the expression levels of pro-BDNF and BDNF were increased by the LSS operation, but administration of SHINBARO2 or E_2_ lowered the LSS-associated upregulation of pro-BDNF and BDNF as analyzed by western blot (Figure 6(c)).

In a BDNF-mediated signaling pathway, the binding of BDNF to tyrosine kinase receptor-B (TrkB) triggers the activation of mitogen-activated protein kinase kinase (MEK) and extracellular signal-regulated kinase (ERK) and regulates cAMP response element-binding protein (CREB) activity [23]. As shown in Figure 6(d), MEK and ERK were activated (p-MEK and p-ERK) in the spinal cord tissue of the LSS-operated control group, and the activation was suppressed after administration of SHINBARO2. In addition, CREB was activated via phosphorylation in the spinal cord tissue of the LSS control group, and SHINBARO2 administration suppressed activation of the CREB transcription factor.

## 4. Discussion

SHINBARO2 is a new prescription medicine based on GCSB-5 that has already been studied in various applications, such as anti-inflammation, nerve regeneration, and cartilage protection in osteoarthritis [24]. However, little is known about the effects of SHINBARO2 in LSS. This study was designed to elucidate the effect of SHINBARO2 on anti-inflammation and pain relief in an LSS model and to further investigate the underlying mechanisms of action.

The common method used in LSS rat models is to insert materials including silicone or wire which narrow the spinal canal and press on the spinal cord to mimic the etiology of radiculopathy. Therefore, LSS is highly related to compression, chemical insult, and vascular and nutritive insufficiency. In this study, we established a chronic spinal nerve root compression model which mimics LSS using a silicone tube. One of the most important parts of this experiment is the way to insert a silicon into the spinal canal. After only a minimal incision to the back of the rat, a syringe containing a silicon tube was inserted through the ligamentum flavum, and only an empty syringe was later removed from the spine of the rat. This method will give an advantage with the less severe tissue damage than surgeries described in previous studies which incise or remove parts of the spine. Since this method is able to reduce unnecessary tissue damage, the inflammatory response in this model is mainly due to LSS, not the surgical procedure.

Moreover, an objective behavioral assessment method is also essential for animal models that evaluate the onset and improvement of LSS. The symptoms, such as neurogenic intermittent claudication, leg pain, tingling, weakness, or insensitivity from the lower back into the buttocks and legs, are the main criteria for judging LSS. However, the degree of these symptoms and pain is not always the same with the degree of imaging LSS. Therefore, the in vivo LSS rat model will give a benefit with the similarity of symptoms of patients compared to the in vitro models.

Specifically, the success of the surgery mimicking LSS is demonstrated by the behavioral assessment of the rats. First, the number of footsteps of the rats in the LSS-operated control group in the running wheel was decreased significantly compared to that of the normal control group, whereas administration of SHINBARO2 effectively recovered these impairments (Figure 2(a)). In addition, SHINBARO2 improved the sensory disturbance caused by LSS as detected with the hot plate method. The surgically induced crippling behavior on thermal stimulation significantly improved in a dose-dependent manner in rats administered SHINBARO2 over two weeks (Figure 2(b)). Furthermore, SHINBARO2 protected against LSS-associated decreases in function without any significant toxicity, as evidenced by body weight changes (Figure 2(c)). Taken together, these data suggest that oral or intraperitoneal administration of SHINBARO2 preserves the spinal structure and effectively prevents functional loss and pain without any significant toxicity.

Because the overproduction of NO and PGE_2_ is highly related to various pathological conditions such as inflammation, the regulation of iNOS and COX-2 is a crucial target for the treatment of inflammatory disorders. The present study was designed to investigate the relation between pain, one of the major symptoms of LSS patients, and inflammation. LSS surgery in the rat model induced the expression of proinflammatory enzymes such as iNOS and COX-2, which were significantly downregulated by the oral or intraperitoneal administration of SHINBARO2 (Figure 3). These inhibitory effects were accompanied by dose-dependent decreases in the protein and mRNA levels of these enzymes in serum, indicating that the inhibition of NO and PGE_2_ levels by SHINBARO2 was attributed to the suppression of the iNOS and COX-2 expression at the transcriptional and translational levels. In addition, the production or function of IL-1*β* and TNF-*α*, proinflammatory cytokines associated with pain and inflammation, plays an important role in many inflammatory responses [25, 26]. In the current study, IL-1*β* and TNF-*α* were significantly elevated upon surgical induction of LSS, and SHINBARO2 was found to effectively suppress those inflammatory responses (Figure 4).

BDNF is a neurotrophin factor that plays an important role in promoting neurogenesis and synaptic plasticity [27]. BDNF acts as a neuromodulator under inflammatory conditions [28], and increased BDNF is associated with a variety of painful conditions [29–32]. Previous studies reported that the expression of BDNF is increased in dorsal root ganglia in a rat LSS model [33]. In the present study, the mRNA level of BDNF was increased by LSS surgery and decreased by SHINBARO2 administration (Figure 6(a)). Additionally, pro-BDNF and BDNF protein expressions were activated by LSS operation and were inhibited by SHINBARO2 treatment. Pro-BDNF is the initially synthesized form of BDNF, which is subsequently cleaved to generate mature BDNF [34]. There is new insight into the mechanism of those neurotrophins, the so-called “yin and yang effect.” Pro-BDNF and BDNF provoke opposite biological effects by activating two distinct receptor systems [35]. The interaction of mature BDNF with Trk receptors leads to cell survival, whereas binding of pro-BDNF to pan-neurotrophin receptor p75 leads to apoptosis [36–38].  Figure 6 shows that both pro-BDNF and BDNF were activated by LSS surgery in spinal cord tissue. However, it could be inferred from the comparison between the LSS-induced groups that the neuroprotective effect of SHINBARO2 was induced by the effects of the reduction in pro-BDNF rather than BDNF.

The MAPK signaling pathway also plays a crucial role in mediating the induction of proinflammatory cytokines [39, 40]. In the present study, SHINBARO2 effectively modulates the MEK-ERK signaling pathway, the downstream signaling of BDNF, rather than JNK and p38 signalings (Figure 6(d)). Collectively, the treatment of SHINBARO2 might modulate the expression of MEK-ERK-CREB axis in the LSS rat model.

## 5. Conclusions

In conclusion, the current findings support the scientific basis of SHINBARO2 that is used clinically. Through the establishment of the LSS rat model, SHINBARO2 has been proven to be effective in reducing LSS symptoms, such as pain and behavior disorders, which are similar to the symptoms observed in actual LSS patients. The reduction of these symptoms by SHINBARO2 is associated with the inhibition of inflammatory mediators, proinflammatory cytokines, and neurotrophic factors. Further research should be conducted to demonstrate the effectiveness of SHINBARO2 in clinical and preclinical settings.

## Figures and Tables

**Table 1:** Sequences of target gene-specific primers used in real-time RT-PCR.

Target genes	Sequences	
Rat iNOS	Sense	5′- ACCATGGAGCATCCCAAGT-3′
	Antisense	5′- CAGCGCATACCACTTCAGC-3′

Rat COX-2	Sense	5′- CTACACCAGGGCCCTTCC-3′
	Antisense	5′- TCCAGAACTTCTTTTGAATCAGG-3′

Rat TNF-*α*	Sense	5′- AGTTGGGGAGGGAGACCTT-3′
	Antisense	5′- CATCCACCCAAGGATGTTTAG-3′

Rat IL-1*β*	Sense	5′- TGTGATGAAAGACGGCACAC-3′
	Antisense	5′- CTTCTTCTTTGGGTATTGTTTGG-3′

Rat BDNF	Sense	5′- CAGCTTGTATCCGACCCTCT -3′
	Antisense	5′- TCCTCTGGAGGATGCCTAAA -3′

Rat *β*-Actin	Sense	5′- CCCGCGAGTACAACCTTCT-3′
	Antisense	5′- CGTCATCCATGGCGAACT-3′

## Data Availability

The data used to support the findings of this study are included within the article.
